# Prey contaminated with neonicotinoids induces feeding deterrent behavior of a common farmland spider

**DOI:** 10.1038/s41598-019-52302-6

**Published:** 2019-11-04

**Authors:** Stanislav Korenko, Pavel Saska, Kristýna Kysilková, Milan Řezáč, Petr Heneberg

**Affiliations:** 1 Czech University of Life Sciences Prague, Faculty of Agrobiology, Food and Natural Resources, Prague, Czech Republic; 2 Crop Research Institute, Functional Biodiversity Group, Prague, Czech Republic; 3 Charles University, Third Faculty of Medicine, Prague, Czech Republic

**Keywords:** Entomology, Animal behaviour, Environmental impact

## Abstract

Neonicotinoids are thought to have negligible repellent or anti-feeding effects. Based on our preliminary observations, we hypothesized that the contamination of spider prey with commonly used neonicotinoids has repellent or feeding deterrent effects on spiders. We tested this hypothesis by providing prey treated or not with field-realistic concentrations of neonicotinoids to the spiders and determining the number of (a) killed only and (b) killed and eaten prey. We exposed adult freshly molted and starved *Pardosa agrestis*, a common agrobiont lycosid species, to flies treated with neonicotinoids (acetamiprid, imidacloprid, thiacloprid and thiamethoxam) at field-realistic concentrations or with distilled water as a control. There were no effects of the exposure of the prey to neonicotinoids on the number of flies captured. However, the spiders consumed less of the prey treated with neonicotinoids compared to the ratio of control prey consumed, which resulted in increased overkilling (i.e., killing without feeding). In female *P*. *agrestis*, the overkilling increased from only 2.6% of control flies to 25–45% of neonicotinoid-treated flies. As the spiders avoided consuming the already captured neonicotinoid-treated prey, the sublethal effects of neonicotinoids extend beyond the simple attractivity/deterrence of the prey itself. The present study demonstrated that prey overkilling serves as a physiological response of spiders to the contact with the prey contaminated with agrochemicals. We speculate that primary contact with neonicotinoids during prey capture may play a role in this unexpected behavior.

## Introduction

Neonicotinoids are compounds that act as potent insecticides but are thought to have negligible repellent or anti-feeding effects. Nevertheless, Kessler *et al*.^1^ published that major pollinators prefer food containing neonicotinoid insecticides when applied at concentrations ranging from 1 nM to 100 nM in sucrose solution. However, the experimental data obtained by other research groups and/or using different model organisms are conflicting. As indicated correctly by Easton and Goulson^2^, experimental confirmation is needed to show whether the food containing neonicotinoids is indeed avoided or preferred and whether this applies equally to spiders, dipterans, beetles, bees, etc. or whether there are taxon-specific responses. They found that pan traps with sucrose contaminated by 0.1 mg mL^−1^ or 0.01 mg mL^−1^ imidacloprid attracted more Lycosidae spiders than those with pure sucrose^2^. As the spiders do not feed on sucrose, they speculated that the attraction of spiders to sites treated with neonicotinoids, where the insects were killed by the insecticides, might result in a situation where the spiders would find themselves in places with few insect prey^2^.

Repellency is often difficult to distinguish from toxicity because the behavioral changes in response to insecticide exposure can be caused by intoxication, chemoreception or a combination of both of these processes^3^. In addition, repellency may prevent an insect from entering treated areas and acquiring a lethal dose of the tested compound^4^. Kinetic response or nondirectional movements can reflect both neurotoxicity and detection, and both manifest as a general increase in locomotor activity^5,6^. As stated by Pekár and Haddad^7^, a selective pesticide should ideally cause no repellency to beneficial arthropods. In the other words, beneficial predators should not be repelled, and their behavior, such as prey capture rate, should not be negatively affected in order to protect their ability to retain their biological control function^7^. Chemosensitive setae of spiders are located at distal and ventral sides of tarsi of their first legs^8^; therefore, the spiders are able to identify at least some residues immediately after touching the contaminated prey. Previously, spiders were shown to avoid surfaces contaminated with fresh (but not 24h-old) residues of the organophosphate phosalone (Zolone 35EC)^7^, a pyrethroid permethrin (Ambush 25EC)^7^, chlorpyrifos and cypermethrin mixture^9^, deltamethrin^9^, and glyphosate^10^ (cf. Michalková and Pekár^11^ for contradictory data); some species avoided even *Bacillus thuringiensis* (Novodor SC) residues^7^. Concening natural products, repellent effects were reported for β-caryophyllene and nerolidol in *Pisaura mirabilis*^12^, nicotin in wolf spiders^13^, chestnuts in multiple spider species^14^, neem (*Azadirachta indica*) seed oil in multiple spider species^15^, and mint oil in *Latrodectus geometricus*^14^. In some cases, the repellence (or toxicity itself) could be caused by additives present in pesticide formulations^16^. The repellency of additives is of use as a potential prevention of ingestion of the pest contaminated by active compounds that are harmful to potential predators or scavengers. Mechanistically, spiders that actively attempt to avoid contact with repellent residues display higher locomotion, avoidance of contaminated surfaces^7^, building their webs distant from contaminated surfaces^7^, or increased dispersal^17^. Among the repellent effects of pesticides on spides that were summarized by Pekár^18^ and in later publications, there were no data on repellent effects of the pesticide contamination of the prey. Nevertheless, the spiders that are specialized for capturing ants are repelled by formic acid, which causes up to 100% overkilling of the prey^19^. This repellent effect is, however, only temporary and the spiders return to their prey and consume it later^20^. Repellency of neonicotinoids has been previously reported from multiple species of insects and other invertebrates (Table 1), but experiments that involved neonicotinoids and claimed the absence of repellence were also reported (Table 2).

**Table 1:** Previously reported repellent effects on insects and other invertebrates in response to neonicotinoids.

Compound	Species (classification)	Note	Reference
Acetamiprid	*Reticulitermes flavipes* (Blattodea)	Confounding effects of paralysis cannot be excluded	^49^
Acetamiprid at ≥ 1 ppm	*Reticulitermes hesperus* (Blattodea)	Confounding effects of paralysis cannot be excluded	^50^
Clothianidin at 10 μg L^−1^	*Bombus terrestris* (Hymenoptera)	Sucrose consumption reduced	^51^
Imidacloprid	*Somaticus terricola* (Coleoptera)	Avoidance of treated areas	^52^
Imidacloprid	*Bemisia argentifolii* (Hemiptera)	Avoidance of treated areas	^53^
Imidacloprid	*Diaphorina citri* (Hemiptera)	Increased dispersal from treated citrus plants; effects were delayed and stemmed rather from feeding deterrence than from immediate repellency	^54^
Imidacloprid at 10 and 100 μg L^−1^	*Bombus terrestris* (Hymenoptera)	Reversible dose-dependent reduction in sucrose consumption	^51^
Imidacloprid	*Gammarus pulex* (Amphipoda)	Antifeedant	^55^
Imidacloprid	*Chironomus riparius* (Diptera)	Antifeedant	^56^
Imidacloprid	*Epeorus longimanus* (Ephemeroptera)	Antifeedant	^57^
Imidacloprid	*Lumbriculus variegatus* (Lumbriculida)	Antifeedant	^57^
Imidacloprid	*Myzus persicae* (Hemiptera)	Antifeedant	^58^
Imidacloprid	*Bemisia tabaci* (Hemiptera)	Antifeedant	^59^
Imidacloprid	*Anoplophora glabripennis* (Coleoptera)	Antifeedant	^60^
Imidacloprid	*Plectrodera scalator* (Coleoptera)	Antifeedant	^60^
Imidacloprid	*Serangium japonicum* (Coleoptera)	Antifeedant	^61^
Imidacloprid	aphids	Antifeedant	^58^
Imidacloprid at 1–0.01 mg L^−1^	Diptera	Decreased captures into yellow pan traps	^2^
Imidacloprid at 1 mg L^−1^	Coleoptera	Decreased captures into yellow pan traps	^2^
Thiacloprid	*Tersilochus obscurator* (Hymenoptera)	Repelled	^62^
Thiamethoxam	*Bactericera cockerelli* (Hemiptera)	Repelled	^63^
Thiamethoxam	*Diaphorina citri* (Hemiptera)	Increased dispersal from treated citrus plants; effects were delayed and stemmed rather from feeding deterrence than from immediate repellency	^54^

**Table 2:** Previously reported absence of repellent effects on insects and other arthropods in response to neonicotinoids.

Compound	Species (classification)	Reference
Clothianidin 1 μg L^−1^	*Bombus terrestris* (Hymenoptera)	^51^
Imidacloprid ≤ 250 ppm	*Macrotermes gilvus* (Blattodea)	^64^
Imidacloprid ≤ 250 ppm	*Reticulitermes flavipes* (Blattodea)	^65^
Thiacloprid, 5 ppm in sucrose solutions	*Apis mellifera carnica* (Hymenoptera)	^66^
Thiamethoxam	*Agriotes obscurus* (Coleoptera)	^67^
Thiamethoxam	*Bombus terrestris* (Hymenoptera)	^51^
Thiamethoxam	*Bemisia tabaci* (Hemiptera)	^68^
Thiamethoxam	*Reticulitermes flavipes* (Blattodea)	^49, 69^
Thiamethoxam	*Comtotermes formosanus* (Blattodea)	^69^
Clothianidin, imidacloprid, or thiamethoxam, from 0.5 nM to 150 nM	*A*. *mellifera* and *B*. *terrestris* (Hymenoptera)	^1^
Imidacloprid, from 1.0 to 0.01 mg L^−1^	Araneae	^2^

Previous studies hypothesized that spiders often adapt to food-limited environments by overkilling their prey^21–24^. Overkilling is also termed wasteful killing, or unnecessary killing – all these terms refer to the killing of the prey without subsequent feeding or discarding partially consumed prey^25^. Overkilling was repeatedly demonstrated as a feeding strategy of cursorial generalist spiders but occurs in specialized spider species as well. Overkilling positively correlates with prey density^19,23,26,27^. The reasons for the use of overkilling as a feeding strategy of spiders are unknown. The neural-constraints hypothesis claims that generalists make poorer decisions than specialists when selecting prey and therefore achieve a higher level of satiation from a single prey item and reduce their subsequent foraging activity^28–30^. This hypothesis expects that such a strategy minimizes the ingestion of noxious chemicals from unsuitable, i.e., noxious, prey^28^. This could be highly relevant in the context of insecticide-treated prey experiments that were performed in the present study. Another hypothesis stated that overkilling is a result of increased aggressivity of certain individuals (or species), which then engage in higher levels of wasteful killing^21,31^. Despite the causes of overkilling are unknown, overkilling, together with delayed saturation of prey capture rates, and partial feeding are responsible for a high asymptote of capture frequency of prey by spiders compared to other predators^32^. To our knowledge, there are no data concerning the effects of chemical compounds on overkilling rates in spiders.

Spiders are among the most abundant predators in agroecosystems, where they are considered major biological control agents of moths, psyllids, aphids, planthoppers and other economically important organisms with the potential to cause adverse effects on crop yields^33–37^. Therefore, we hypothesized that the neonicotinoids that are commonly used in agriculture (acetamiprid, imidacloprid, thiacloprid and thiamethoxam) have repellent or feeding deterrent effects on spiders. We tested this hypothesis by providing prey treated or not with field-realistic concentrations of neonicotinoids to the spiders and determined the number of a) killed prey, b) killed and eaten prey, and c) overkilled prey that was killed but left uneaten.

## Materials and Methods

As a model, we used the lycosid spider *Pardosa agrestis* (Westring, 1861) (Araneae: Lycosidae). This spider actively searches for prey on the ground. We collected juveniles of *P*. *agrestis* (n = 280) from barley fields in the Tursko, Czechia (50.11°N, 14.19°E, 300 m a.s.l.) environment in April and May 2017. We kept the spiders individually in glass tubes (diameter 15 mm, length 60 mm) with a layer of plaster of Paris at the bottom. We moistened the plaster of Paris with a few drops of water at three-day intervals to maintain adequate humidity. We kept the spiders at 20–22 °C under a photoperiod of 16 h light/8 h darkness. We used wingless *Drosophila melanogaster* Meigen, 1830 (Diptera: Drosophilidae) flies and juvenile *Acheta domestica* (Linnaeus, 1758) (Orthoptera: Grillidae) crickets as food during rearing to adulthood.

When spiders reached adulthood, we corroborated their species identity according to Nentwig *et al*.^38^. The average body size (prosoma and opisthosoma length) displayed sex-specific differences, with females being longer (6.16 ± 0.09 mm) than males (5.71 ± 0.08 mm) (Mann-Whitney U test U = 798.5, *p* = 0.002, n = 50 in each group). We took freshly molted individuals with no signs of harm and split them into five experimental groups of 20 individuals each. Each experimental group contained ten females and ten males. During the experiments, we fed spiders with flies treated with insecticides or with distilled water as a control (mock). The tested insecticides consisted of the neonicotinoids acetamiprid (formulated as Mospilan 20 SP; dilution 7.32 μg L^−1^; treatment 3.9 mg cm^−2^), imidacloprid (Confidor 200 OD; dilution 73.2 μg L^−1^; treatment 1.7 mg cm^−2^), thiacloprid (Biscaya 240 OD; dilution 24.4 μg L^−1^; treatment 4.5 mg cm^−2^) and thiamethoxam (Actara 25 WG; dilution 8.54 μg L^−1^; treatment 3.7 mg cm^−2^). Thus, we applied all the neonicotinoids in dilutions recommended by their manufacturers for use in spraying crops to eliminate pest insects.

Before the experiments, we starved the adult spiders for 10 days. We performed the experiments under controlled laboratory conditions (shaded room with a natural day/night regimen, temperature 24 ± 1 °C). We placed the standardized spiders (reared to adulthood and starved) individually into Petri dishes (10 mm tall and 50 mm in diameter) with a layer of wet filter paper on the bottom to maintain humidity during the experiment. We allowed the spiders to settle for 10 min to become acclimated to the experimental arena. We then provided the spiders with wingless *D*. *melanogaster* that were treated with neonicotinoids or distilled water. Polyphagous predators, including *Pardosa* spiders, readily accept *Drosophila* flies^39^. We provided each spider with three flies that were simultaneously present in a Petri dish. We recorded the number of killed flies in six periods, each lasting 30 min. We distinguished the prey that was only killed from the prey that was killed and eaten, i.e., sucked out. During every control, we replaced any dead flies with live flies, aiming to maintain a constant density of prey in the Petri dish. We excluded spiders that did not accept prey and molted within 24 h after the experiment (n = 1) from further analyses to avoid the effect of feeding cessation, i.e., a behavior known to be displayed by spiders before molting^40^. We measured the body size of each spider and analyzed possible relationships between spider size, prey treatment and feeding deterrent behavior expressed as changes in the number of captured prey, the number of fed prey and the number of overkilled prey. Data are shown as the mean ± SE unless stated otherwise. We used generalized linear model (GLM) with a Poisson structure of errors and a log link function to test for the differences in killing behavior of spiders between treatments. We included the insecticide treatments, sex and their interaction as explanatory variables and the total number of killed flies, the number of killed flies from the first batch, or the number of killed but not consumed flies as response variables, each fitted in separate models. We used quasi-Poisson distribution when overdispersion appeared to be significant according to the overdispersion test from the R package AER^41^. We assessed the significance of individual terms by deletion tests. We conducted the analysis in the R environment, version 3.1.2^42^.

## Results

**Figure 1:**
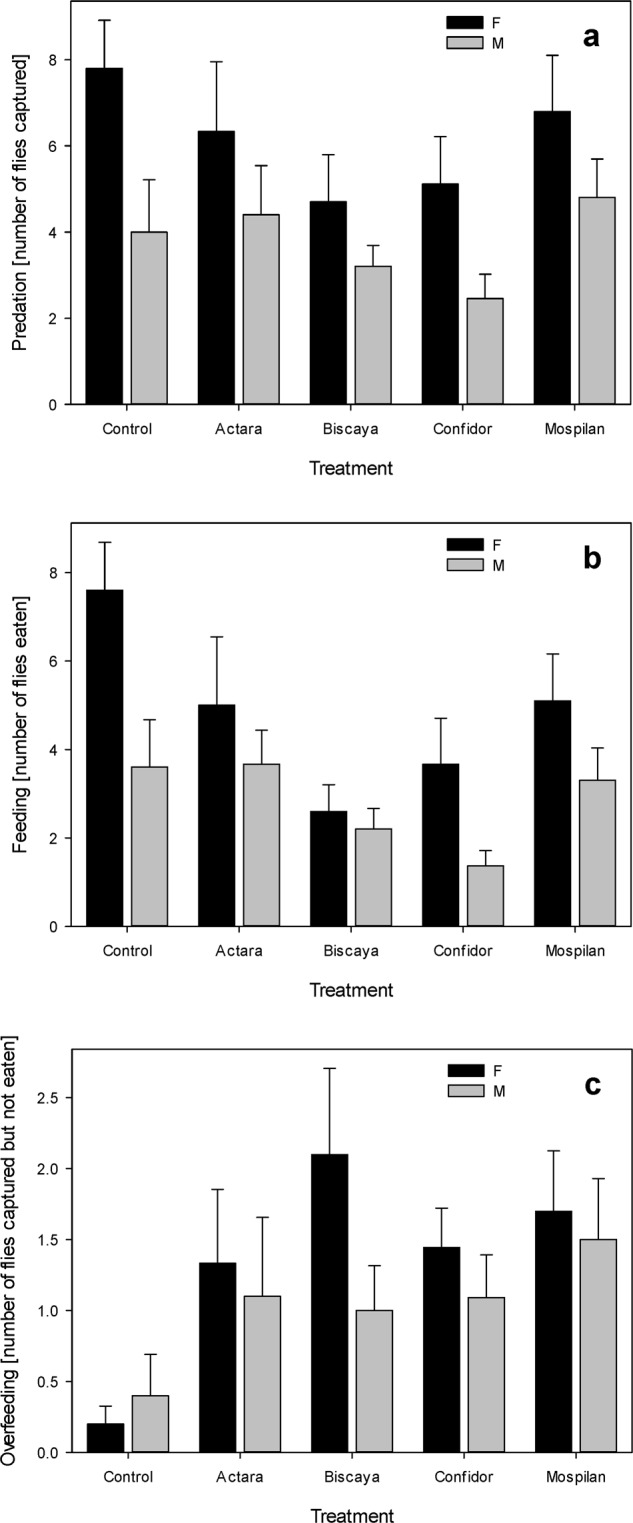
Effects of the treatment of prey with field-realistic concentrations of neonicotinoids on the number of flies captured only (**a**), captured and eaten (**b**), and overkilled (**c**).

Mock-treated females captured more prey than did males (Fig. 1a). The initial GLM model that tested for the differences in total consumption between treatments, using Poisson distribution, revealed overdispersion (z = 3.6, *p* = 0.00016). We therefore applied the quasi-Poisson model, which confirmed the significant contribution of sex but not the neonicotinoid treatments to the predation rates (Table 3). The mean predation rate of females was 5.42 (95% CI 4.49–6.53); the mean predation rate of males was 3.31 (95% CI 2.63–4.18).

**Table 3:** Effect of neonicotinoid treatment and sex on the predation of fruit flies by *Pardosa agrestis*. Total predation was compared using GLM-quasi-Poisson and predation of the first provided batch and overkilling using GLM-Poisson. Terms significant at α = 0.05 are in bold.

Term	D_f_	Deviance	Resid. D_f_	Residual deviance	*P*
**Total predation**					
Null			98	256.25	
Treatment	4	12.40	94	243.85	0.27
Sex	1	24.83	93	219.02	**0**.**002**
Treatment:Sex	4	3.75	89	215.27	0.81
**Predation of the first provided batch of flies**					
Null			98	140.13	
Treatment	4	8.60	94	131.54	0.07
Sex	1	5.74	93	125.80	**0**.**02**
Treatment:Sex	4	0.62	89	125.18	0.96
**Overkilling**					
Null			98	155.10	
Treatment	4	21.63	94	133.47	**0**.**0002**
Sex	1	2.64	93	130.84	0.10
Treatment:Sex	4	3.96	89	126.88	0.41

To avoid any effect of satiation, we also tested separately the effects of treatments and sex on predation of the first batch of flies provided (three flies provided simultaneously). The initial GLM model for Poisson distribution fit the data well, with no significant overdispersion present (z = −0.04, *p* = 0.52). The Poisson model revealed a significant contribution of sex, but the effect of treatments was again not significant (Table 3). The mean predation rate of females was 1.71 (95% CI 1.38–2.12); the mean predation rate of males was 1.12 (95% CI 0.86–1.45).

However, prey treated by Confidor and Biscaya were eaten less compared to control prey (Fig. 1b). Therefore, we tested the effects of treatments and sex on overkilling, i.e., the number of killed but not eaten prey items. In female *P*. *agrestis*, the overkilling increased from only 2.6% of control flies to 21.1% (Actara) – 44.7% (Biscaya) of neonicotinoid-treated flies, depending on the tested compound. In male *P*. *agrestis*, the overkilling increased from 10.0% of control flies to 25.0% (Actara) – 44.4% (Confidor) of neonicotinoid-treated flies. The initial GLM model for the Poisson distribution of the data fit well, with no significant overdispersion present (z = 1.54, *p* = 0.06). In contrast to the effects on predation, the Poisson model revealed a significant contribution of the treatments but not the effect of sex (Table 3; Fig. 1c). The analysis revealed that the spiders offered with the control diet exhibited significantly lower overkilling (z = −3.540, *p* = 0.0004) than those offered prey treated with any of the four neonicotinoids (Table 3). The mean overkilling by control spiders was 0.20 individuals (95% CI 0.08–0.49); the mean overkilling by spiders fed with neonicotinoid-treated prey was 1.27 individuals (95% CI 1.04–1.54) and did not differ among the tested compounds (Tables 3 and 4).

**Table 4:** Predicted mean values and 95% confidence intervals of overkilling by *Pardosa agrestis* under different treatments of neonicotinoids. Prediction was based on GLM-Poisson.

Treatment	Predicted mean	95% CI
Actara	1.11	0.72–1.70
Biscaya	1.18	0.66–2.09
Confidor	1.13	0.63–2.02
Mospilan	1.27	0.72–2.23
Control	0.23	0.09–0.60

## Discussion

We provided the first evidence of feeding deterrent behavior of common farmland spiders in response to prey that is contaminated by neonicotinoids because the frequency of killed but not eaten prey increased in insecticide-treated groups, although the total and initial predation did not differ. Therefore, the present study confirmed previous speculation by Řezáč *et al*.^43^, who claimed in their study on the effects of Mospilan and other pesticides on the functional response of spiders that “Although we have not studied whether all of the killed prey was consumed, it is likely that *P*. *cespitum* performed overkilling”. Therefore, the part of the initial hypothesis that predicted the feeding deterrent behavior of spiders in response to the treatment of their prey with neonicotinoids was supported with outcomes of the above-described experiments.

On the other hand, we did not observe that the tested spiders were repelled from the prey killing. Therefore, the neonicotinoids did not provide a sort of chemical protection to the prey and did not cause any avoidance of the prey by the spiders. The neonicotinoids also did not increase the attractiveness of the prey, as would be expected from the study by Easton and Goulson^2^, who demonstrated that spiders were attracted to yellow pan traps with aqueous glucose solution of imidacloprid at 0.01–1 μg L^−1^.

Except for the abovementioned paper by Easton and Goulson^2^, the data on feeding deterrence of neonicotinoids are absent for spiders and are available mostly for honeybees and bumblebees. However, even the physiological experiments on honeybees did not provide an unanimous concensus concerning the deterrence of bees. In some studies, different neonicotinoids displayed opposite effects^44,45^. In other studies, neonicotinoids generally stimulated feeding but signaling from gustatory neurons or sucrose-sensitive neurons was not detectable, suggesting no taste of neonicotinoids and no repellence effect in honeybees and bumblebees^1^. Moreover, the results of field studies were inconsistent and were likely affected by the use of insufficiently characterized “pesticide-free” control areas^46,47^.

Combined, the inconsistency of available data makes it difficult to speculate on the causes of the feeding deterrent behavior of spiders in response to neonicotinoids. It is possible that the first contact with neonicotinoids during the prey capture affected the health of the spiders. Řezáč *et al*.^48^ recently reported that contact with neonicotinoids often leads to the temporary paralysis of spiders. We therefore speculate that the lack of taste and repellence effects causes prey contaminated with neonicotinoids to be captured equally to untreated prey. However, the initial contact with the prey causes adverse health effects that deter the spider from the consumption of already captured prey. The paralysis that is induced by neonicotinoids might actually be behind the seemingly increased number of spiders captured in the seminal study by Easton and Goulson^2^. During pan trapping experiments, most spiders actually manage to escape unless the pans are deep enough to prevent such behavior. However, if paralysis plays a role, the spiders would be unable to escape; therefore, they would be captured at higher rates compared to control pan traps even in the absence of attractiveness of the neonicotinoids themselves.

In conclusion, we substantially broadened the knowledge on the sublethal effects of neonicotinoids in spiders. As the spiders avoided consuming the already captured prey, the sublethal effects of neonicotinoids extend beyond the simple attractivity/deterrence of the prey itself. We found that spiders behaved as proposed previously for the contact with noxious prey. Overkilling of the prey, which increased substantially when we provided the spiders with the prey contaminated with neonicotinoids, was previously hypothesized to minimize the ingestion of noxious naturally present chemicals from unsuitable prey^28^. We demonstrated that increased prey overkilling serves as a physiological response of spiders to the contact with the prey contaminated with agrochemicals. Neurophysiological studies are needed to elucidate whether the observed feeding deterrent behavior was caused by the reception of the neonicotinoids or, more likely, by effects of the primary contact with neonicotinoids during the prey capture.

## Data Availability

All data generated or analysed during this study are included in this published article.
